# Identification of the Weevil immune genes and their expression in the bacteriome tissue

**DOI:** 10.1186/1741-7007-6-43

**Published:** 2008-10-16

**Authors:** Caroline Anselme, Vicente Pérez-Brocal, Agnès Vallier, Carole Vincent-Monegat, Delphine Charif, Amparo Latorre, Andrés Moya, Abdelaziz Heddi

**Affiliations:** 1 Université de Lyon, INRA, INSA-Lyon, IFR-41, UMR203 BF2I, Biologie Fonctionnelle Insectes et Interactions, F-69621 Villeurbanne, France; 2 Institut Cavanilles de Biodiversitat i Biologia Evolutiva, Universitat de València, Apartado de Correos 22085, 46071 Valencia, Spain; 3 UMR CNRS 5558 Laboratoire de Biométrie et Biologie Evolutive, Université Claude Bernard Lyon, F-69621 Villeurbanne, France

## Abstract

**Background:**

Persistent infections with mutualistic intracellular bacteria (endosymbionts) are well represented in insects and are considered to be a driving force in evolution. However, while pathogenic relationships have been well studied over the last decades very little is known about the recognition of the endosymbionts by the host immune system and the mechanism that limits their infection to the bacteria-bearing host tissue (the bacteriome).

**Results:**

To study bacteriome immune specificity, we first identified immune-relevant genes of the weevil *Sitophilus zeamais *by using suppressive subtractive hybridization (SSH) and then analyzed their full-length coding sequences obtained by RACE-PCR experiments. We then measured immune gene expression in the bacteriome, and in the aposymbiotic larvae following *S. zeamais *primary endosymbiont (SZPE) injection into the hemolymph, in order to consider the questions of bacteriome immune specificity and the insect humoral response to symbionts. We show that larval challenge with the endosymbiont results in a significant induction of antibacterial peptide genes, providing evidence that, outside the bacteriome, SZPE are recognized as microbial intruders by the host. In the bacteriome, gene expression analysis shows the overexpression of one antibacterial peptide from the *coleoptericin *family and, intriguingly, homologs to genes described as immune modulators (that is, *PGRP-LB, Tollip*) were also shown to be highly expressed in the bacteriome.

**Conclusion:**

The current data provide the first description of immune gene expression in the insect bacteriome. Compared with the insect humoral response to SZPE, the bacteriome expresses few genes among those investigated in this work. This local immune gene expression may help to maintain the endosymbiont in the bacteriome and prevent its invasion into insect tissues. Further investigations of the *coleoptericin*, the *PGRP *and the *Tollip *genes should elucidate the role of the host immune system in the maintenance and regulation of endosymbiosis.

## Background

Chronic bacterial infections are widespread in nature and exhibit a large range of interactions with their host, from mutualism to parasitism. In insects, symbiotic intracellular bacteria (endosymbionts) are deeply integrated into host cell biology and development as they are transmitted maternally through hundreds of host generations, and early on in the insect embryogenesis they invade specialized host cells called bacteriocytes that sometimes form a specific organ, the bacteriome [1-3]. Physiological and molecular investigations have provided evidence that endosymbionts supply their host's diet with limiting nutrients, thereby improving their invasive power and their ability to settle on nutritionally poor sources and habitats, such as blood (*Glossina*, *Rhodnius*), plant sap (aphids, psyllids, whiteflies, mealybugs), and cereal grains (*Sitophilus*) [2,4].

However, while the physiological and the evolutionary aspects of insect endosymbiosis have been thoroughly investigated over the past two decades, very little is known about the molecular mechanisms that permit the establishment, then the maintenance and the regulation of such beneficial interactions. One striking question concerns the interaction between the bacteria and the host innate immune system, an area which has been relatively well investigated in pathogenic relationships compared with mutualistic associations that have been recently approached in only a few systems [5-8].

To combat infection, insects rely on multiple innate defense reactions. Insect immunity includes physical barriers, together with local and systemic immune responses involving both cellular and humoral pathways (reviewed in [9]). Activation of the humoral pathway consists of microbe-associated molecular pattern recognition by pattern recognition receptors, such as peptidoglycan recognition proteins (PGRPs), and the activation of intracellular signaling pathways, such as the Toll and the Immune deficiency (Imd) pathways. These pathways activate, in particular, the production and the secretion of a panel of antimicrobial peptides (AMPs) in response to Gram-positive and Gram-negative bacteria. In addition to AMP production, the insect humoral response also involves a proteolytic cascade leading to prophenoloxidase (PPO) activation and subsequent melanin synthesis at the site of cuticular injury. This reaction, called melanization, plays a key role in wound healing, encapsulation, sequestration of microbes and production of toxic intermediates [10]. While the systemic response is by far the best characterized pathway among immune reactions, the local immune response (also known as epithelial immunity) was only recently shown to significantly contribute to protection against invading microorganisms in the alimentary tract and tracheae. In the gut, for example, there is an inducible local production of AMPs. This response is triggered upon natural infection by Gram-negative bacteria and is mediated by the Imd pathway [11]. The gut epithelium also expresses amidase PGRP. It has been shown that these PGRPs, which scavenge peptidoglycan released by commensal bacteria, reduce gut immune reactivity and avoid a state of permanent immune activation in this tissue [12,13].

In a previous work on the mutualistic interaction between the weevil *Sitophilus zeamais *and a γ-proteobacterium called *S. zeamais *primary endosymbiont (SZPE) (see [14] for a review on the model), we discovered the overexpression of a member of the *PGRP *gene family in the bacteriome tissue of the host [15,16]. We showed that *wPGRP *is induced after a bacterial challenge and that *wPGRP *gene expression depends on bacterial growth. Moreover, we have shown that the *wPGRP *gene was induced concomitantly with an endosymbiont release from the bacteriocytes during nymphosis, demonstrating that the *PGRP *gene family is involved in host-symbiont interaction [16].

This study was dedicated to enlarging the panel of insect immune genes involved in the host-symbiont interaction. We first identified the host immune-relevant genes by suppressive subtractive hybridization (SSH), and analyzed *in silico *their full-length coding sequences completed with RACE-PCR. We then measured their steady-state levels in aposymbiotic larvae challenged with SZPE and in the bacteriocyte cells of naturally infected larvae. We show that experimental infection of larvae with SZPE results in a significant induction of AMPs and that only few immune gene transcripts, including homologs to an antibacterial peptide and immunomodulators, are accumulated in the bacteriome tissue. These data reveal that endosymbionts are perceived as intruders while being present in hemolymph and that there is a local immune gene expression in the bacteriome. This study provides the first indication of how insects may maintain endosymbionts within the bacteriome and prevent their invasion into insect tissues.

## Results

### Identification of immune-relevant genes in *Sitophilus zeamais*

As the *Sitophilus *genome has not been sequenced, we applied SSH technology to cDNA from *E. coli*-infected larvae and cDNA from naive larvae to identify the immune genes of interest to this work. To obtain genes expressed at different phases of the immune response, three RNA samples were extracted 3, 6 and 12 hours after *Escherichia coli *infection and mixed prior to cDNA synthesis. We sequenced 485 expressed sequence tags (ESTs) from the subtracted library. Following quality analysis, trimming and chimeric sequence digestion (see *Materials and Methods*), we assembled 475 sequences into 273 putative transcripts, consisting of 62 contigs and 211 singletons (Table 1).

**Table 1:** General characteristics of *Sitophilus zeamais *ESTs from suppressive subtractive hybridization between *E. coli*-infected and naive larvae

Total number of cDNA reads	485
Total number of cDNAs analyzed	475

Average ESTs length (bp)	373

**cDNA assembly^a ^result**	

Number of ESTs in contigs	264

Number of contigs	62

Number of singletons	211

Number of consensus	273

**Redundancy ^b^**	55%

Number of contigs containing	

2 to 4 ESTs	49

5 to 10 ESTs	8

> 10 ESTs	5

**Functional classification**	

No Uniprot ^c ^hits	17%

No GO assignment	37%
GO Biological Process (Level 2)	Number of contigs

*Reproduction*	2

*Immune system process*	6

*Metabolic process*	82

*Cellular process*	71

*Viral reproduction*	2

*Reproductive process*	2

*Biological adhesion*	3

*Multicellular organismal process*	4

*Developmental process*	7

*Growth*	1

*Response to stimulus*	13

*Localization*	26

*Establishment of localization*	26

*Multi-organism process*	1

*Biological regulation*	11

^a. ^TGICL parameters: ESTs with 94% identity over at least 30 base pairs and a maximum length of unmatched overhangs of 30 nucleotides were clustered together.

^b. ^Redundancy = number of ESTs in contigs/Total number of ESTs.

^c. ^UniProt Rel. 10 (SWISS-PROT 52 + TrEMBL 35) April 10, 2007, cut-off E-value was 10.

To gain insight into the function of EST products, we compared the 273 resulting sequences with the UniProt databases and classified them using the Gene Ontology (GO) scheme. Moreover, we constructed a database where all the ESTs and a complete list of BlastX matches, together with their functional classification, can be found. Of these 273 sequences, 83% successfully matched against the UniProt database and 63% were functionally classified (Table 1). Concerning the immune genes, around 12% of the ESTs have similarities with transcripts that are known to encode proteins involved in an immune function. According to sequence identity, some ESTs showed only a weak similarity to antibacterial peptides, such as diptericin A, cecropin A1, sarcotoxin II-1 and luxuriosin. Nevertheless, taking into account their high redundancy (12, 4, 15, and 4 copies, respectively), we have included them in this study. In addition, sequence analysis of ESTs with similarities to diptericin, tenecin and luxuriocin has uncovered various isoforms of these peptides (see Additional file 1). However, due to the high DNA and protein sequence identities (76% to 97% and 91% to 100%, respectively) we have analyzed only one isoform of each gene family in this work.

### Analysis of full-length cDNA of immune genes obtained by RACE-PCR

**Figure 1:**
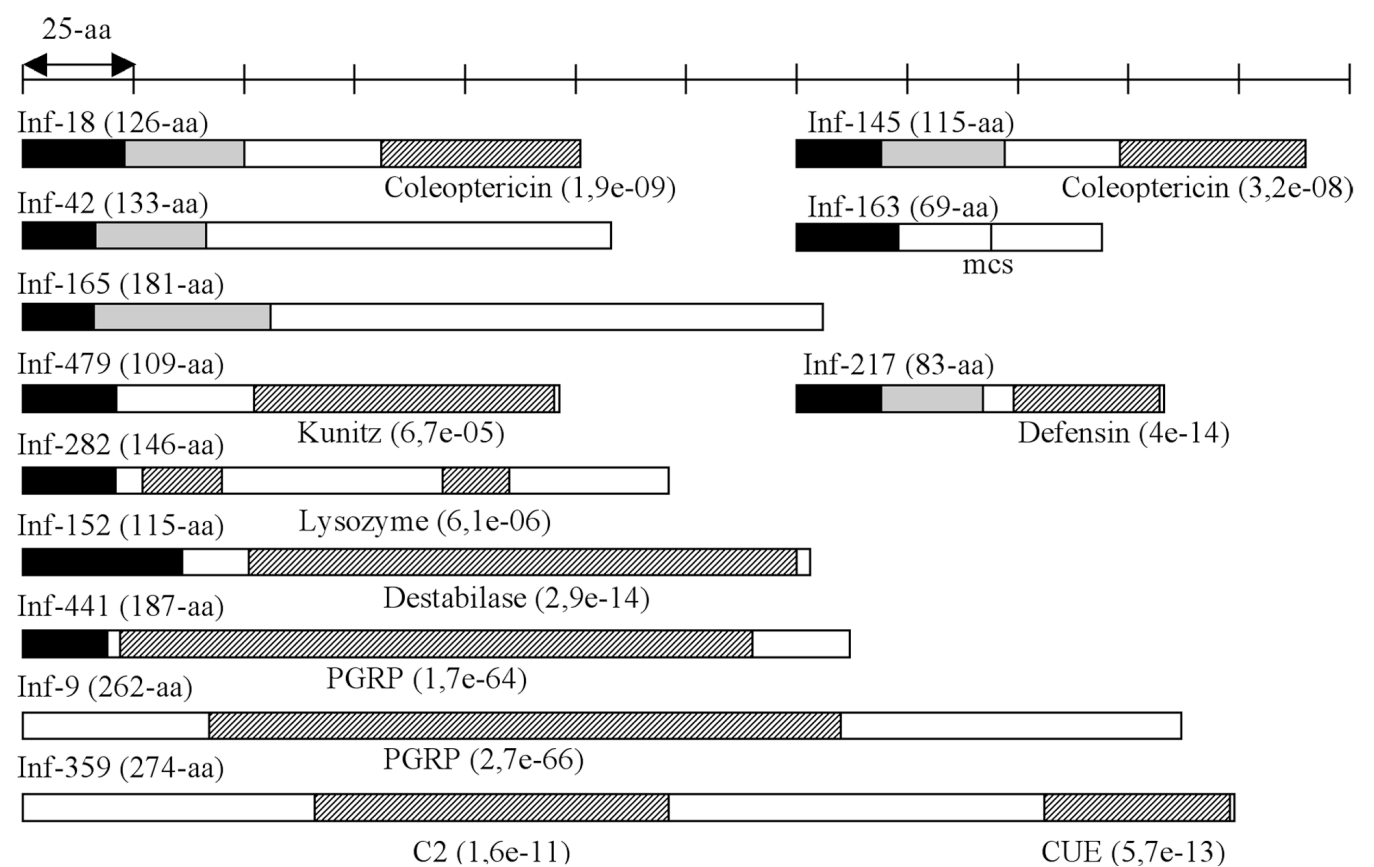
**Schematic representations of putative *Sitophilus zeamais *proteins with similarity to antibacterial peptides, lysozymes, PGRP and Tollip**. For each gene, the whole cDNA sequence was obtained from the corresponding EST by RACE-PCR and was then confirmed by whole cDNA amplification and sequencing. For each cDNA, ORF was predicted using the MacMolly software package. The top scale shows the length of the various domains of the proteins (aa, amino acid). Black regions indicate the predicted signal peptide (TargetP) and gray regions the putative propeptide domain according to the conserved R-x-(K/R)-R motif. mcs: minimal cleavage site corresponding to an R-x-x-R motif with an additional arginine in P6 position, which may enhance cleavage. Regions with similarity to conserved domains detected by InterProScan are indicated as hatched regions with the associated E-value. Coleoptericin, IPR009382; Defensin, IPR001542; Kunitz, IPR002223; Lysozyme, IPR000974; Destabilase, IPR008597; PGRP, IPR002502; C2, IPR000008; CUE, IPR003892. The accession numbers of the complete coding sequences are: INF-18, EU282111; INF-145, EU282117; INF-42, EU282112; INF-163, EU282118; INF-165, EU282113; INF-479, EU282119; INF-217, EU282115; INF-282, EU282114; INF-152, EU282120; INF-441, EU282121; INF-9, EU282122; INF-359, EU282116.

To improve sequence similarities, we applied the RACE-PCR technology and have sequenced the full cDNA sequences of ESTs (one EST for each cluster) with similarities to antibacterial peptides, lysozymes, PGRPs and Tollip (Figure 1). We also analyzed the cDNA sequences *in silico *to identify conserved protein domains and to predict the cellular localization of the peptides and proteins.

#### Antimicrobial peptides

AMPs typically contain fewer than 100 amino acid residues, divided in most cases into three domains: a signal peptide, a propeptide (R-x-(K/R)-R motifs), and the mature peptide. The first two domains are proteolytically cleaved to release the mature peptide active against bacteria [17-19].

A signal peptide and an R-x-(K/R)-R motif have been predicted for all the putative antibacterial peptides of *S. zeamais*, except for INF-163 and INF-479, for which only a signal peptide is reliably predicted (Figure 1). According to protein similarity, we confirmed the identification of two AMPs (INF-18 and INF-145, 40% identity) belonging to the coleoptericin family, a defensin with sequence identity of 66% (32/48) to tenecin (INF-217), and a homolog of luxuriosin, an AMP characterized by a Kunitz domain [20], with a sequence identity of 42% (45/106). For ESTs INF-42, INF-163 and INF-165, the full-length transcripts still show weak similarities to AMPs and no characteristic domain has been identified for the putative peptides, which cannot be considered as antibacterial peptides without further analysis (Figure 1).

#### Lysozymes

Lysozymes are widespread enzymes characterized by their ability to cleave bacterial peptidoglycans. Among lysozymes, several types have been described according to the genomic structure and phylogenetic data: the c (chicken), g (goose), i (invertebrate), phage, bacteria and plant types [21]. Here, according to sequence homology, we have identified two lysozymes: an i-type (INF-152), with a destabilase-related domain identified in an i-type lysozyme from the leech [22], and a c-type lysozyme (INF-282) with a characteristic lysozyme domain. Both proteins have a predicted signal peptide.

#### PGRP

Two PGRP transcripts were identified in the subtracted library. One EST (INF-9) corresponds to the *wPGRP *gene previously identified [15,16] while the EST INF-441 is an additional weevil *PGRP *gene. These two genes will be referenced as *wPGRP1 *and *wPGRP2*, respectively. RACE-PCR product analysis has shown that the two wPGRP proteins have 30% identity and have both conserved the residues necessary for amidase activity [23]. According to *in silico *analysis, wPGRP1 may be an intracellular amidase while wPGRP2 may be secreted in hemolymph.

#### Tollip (Toll interacting protein)

Among the ESTs of the subtracted library, we found a sequence (INF-359) with homology to Tollip, a regulator of the Toll-like receptors pathway described in mammals [24-26]. Analysis of the RACE-PCR product has confirmed homology to the mammal genes since the predicted protein of *S. zeamais *shares 46% identity (127/275) with the Tollip protein from *Mus musculus*.

### Gene expression analysis after sterile or septic injury of weevil aposymbiotic larvae

To determine genes induced by wounding (pricking effect) and genes specifically induced by bacterial challenge, using qRT-PCR we have compared the transcript levels in aposymbiotic larvae after sterile and septic injury, and in naive aposymbiotic larvae taken as a control (Table 2).

**Table 2:** Immune-related ESTs and comparison of gene expression between naive larvae, mock-infected larvae and larvae challenged with *Escherichia coli*.

**EST**	**Protein description**	**E-value**	**Target Organism**	**UniProt Acc. Num.**	**qRT-PCR Fold change**	
	***Antibacterial peptides***				**sterile injury**	**septic injury**

INF-18	Coleoptericin	3E-15	*Zophobas atratus*	P80032	11.4*	86.1* (*)

INF-42	Diptericin A	2.6	*Glossina morsitans*	Q8WTD5	> 10*	> 300* (*)

INF-145	Acaloleptin A	2E-15	*Acalolepta luxuriosa*	Q76K70	1.8	43.1*

INF-163	Cecropin A1	0.68	*Drosophila mauritiana*	P81685	1	31.5*

INF-165	Sarcotoxin II-1	0.67	*Sarcophaga peregrina*	P24491	0.8	31.6*

INF-217	Tenecin-1	3E-13	*Tenebrio molitor*	Q27023	9.7*	314.9* (*)

INF-479	Luxuriosin	0.18	*Acalolepta luxuriosa*	Q60FC9	2.8*	4.5*

	***Lysozymes***					

INF-152	Lysozyme i-1	1E-05	*Anopheles gambiae*	Q6GU90	7.8*	5.2*

INF-282	Lysozyme C-1	6E-17	*Anas platyrhynchos*	P00705	7.1*	5.4*

	***PGRP***					

INF-9	PGRP sb2	7E-57	*Aedes aegypti*	Q1HRH3	2.3*	6.7* (*)

INF-441	PGRP	9E-38	*Biomphalaria glabrata*	A0T2Q1	7.8*	11.6*

	***Immune regulator***					

INF-359	TOLLIP	3E-48	*Mus musculus*	Q9QZ06	1.2	1.7

	***Phenoloxidase pathway***					

INF-506	PPAF	2E-15	*Holotrichia diomphalia*	Q9GRW0	2.8*	2.7*

INF-74	Serpin-4A	2E-20	*Manduca sexta*	Q6Q2D8	2.8*	2.9*

	***Proteases***^a^					

INF-20	IMPI	2E-11	*Galleria mellonella*	P82176	2.3*	3.4*

INF-91	Cysteine-rich venom-like protein	7E-09	*Aedes albopictus*	Q5MIW2	3.4*	7.5* (*)

INF-258	Pattern recognition serine proteinase	7E-28	*Manduca sexta*	Q69BL0	ND	ND

INF-459	Hemolymph proteinase 17	3E-10	*Manduca sexta*	Q5MPB8	1.7	4.4

INF-515	Trypsin-like serine proteinase	5E-27	*Anthonomus grandis*	Q64ID5	1.4	1.1

	***Cytoskeleton***					

INF-13	profilin	3E-29	*Apis mellifera*	Q6QEJ7	0.9	0.8

-	actin	-	*Sitophilus zeamais*	-	1	1.3

ESTs were analyzed as described in *Materials and Methods *and classified according to gene family or function. Transcripts were quantitated by qRT-PCR in untreated aposymbiotic larvae (control), in larvae six hours after a mock-infection (sterile injury) or an *E. coli *infection (septic injury). The fold-change of gene expression after sterile or septic injury is expressed relative to the transcript levels in untreated larvae. For each sample, the transcript level is estimated from the mean of three independent measurements after normalization with the expression of the *gapdh *gene. The transcript amounts of the genes corresponding to the EST INF-42 and INF-258 are too weak to be quantified in the control larvae and in all the samples, respectively. Comparisons of transcript levels between the three samples were made using nonparametric tests. A significant increase in transcript level after sterile or septic injury is indicated by an asterisk (*p *< 0.05). A significant difference between sterile and septic injury is indicated by a second asterisk (*p *< 0.05). Acc. Num., accession number; ND, non-determined. ^a. ^Proteases can represent either signaling proteins or immune effectors.

According to the Kruskal-Wallis rank sum test, most of the genes with sequence similarity to AMPs are highly induced (30 to 300-fold) after *E. coli *infection, including genes without any significant antibacterial domain (Table 2 and Figure 1). Some peptide genes, such as INF-18, INF-42 and INF-217, are also slightly induced (10-fold) after a sterile injury. INF-479, the homolog of *luxuriosin*, is the only peptide gene induced after sterile injury but not in response to *E. coli *challenge. Finally, both lysozyme genes (INF-152 and INF-282) are induced after sterile injury, independently of the bacterial infection.

The qRT-PCR data show that both *wPGRP *genes are upregulated. However, while *wPGRP1 *(INF-9) is weakly induced by injury (2.3-fold) and more strongly induced after *E. coli *infection (6.7-fold), *wPGRP2 *(INF-441) is induced by sterile injury only (7.8 to 11.6-fold).

As regulators we quantified the expression of genes with similarity to proteases and protease inhibitors, in addition to the *Tollip *gene. The *Tollip *gene (INF-359) and two genes with similarity to proteinases (INF-459 and INF-515) were shown to be constitutively expressed. All the other genes were induced after sterile injury, except the cysteine-rich venom-like proteinase homolog (INF-91) that was induced after infection with *E. coli*. However, no data are available concerning the function of this protein identified in the salivary gland of *Aedes albopictus*.

In addition to humoral immune response genes we quantified two cytoskeleton genes, as the participation of actin cytoskeleton regulation proteins in innate immunity has been established by functional genomic analysis of phagocytosis [27]. However, no variation in the transcript levels has been observed, either for the *actin *gene or for the homolog of *profilin*, an actin polymerization regulator (INF-13).

### Gene expression analysis in aposymbiotic larvae challenged with SZPE

**Figure 2:**
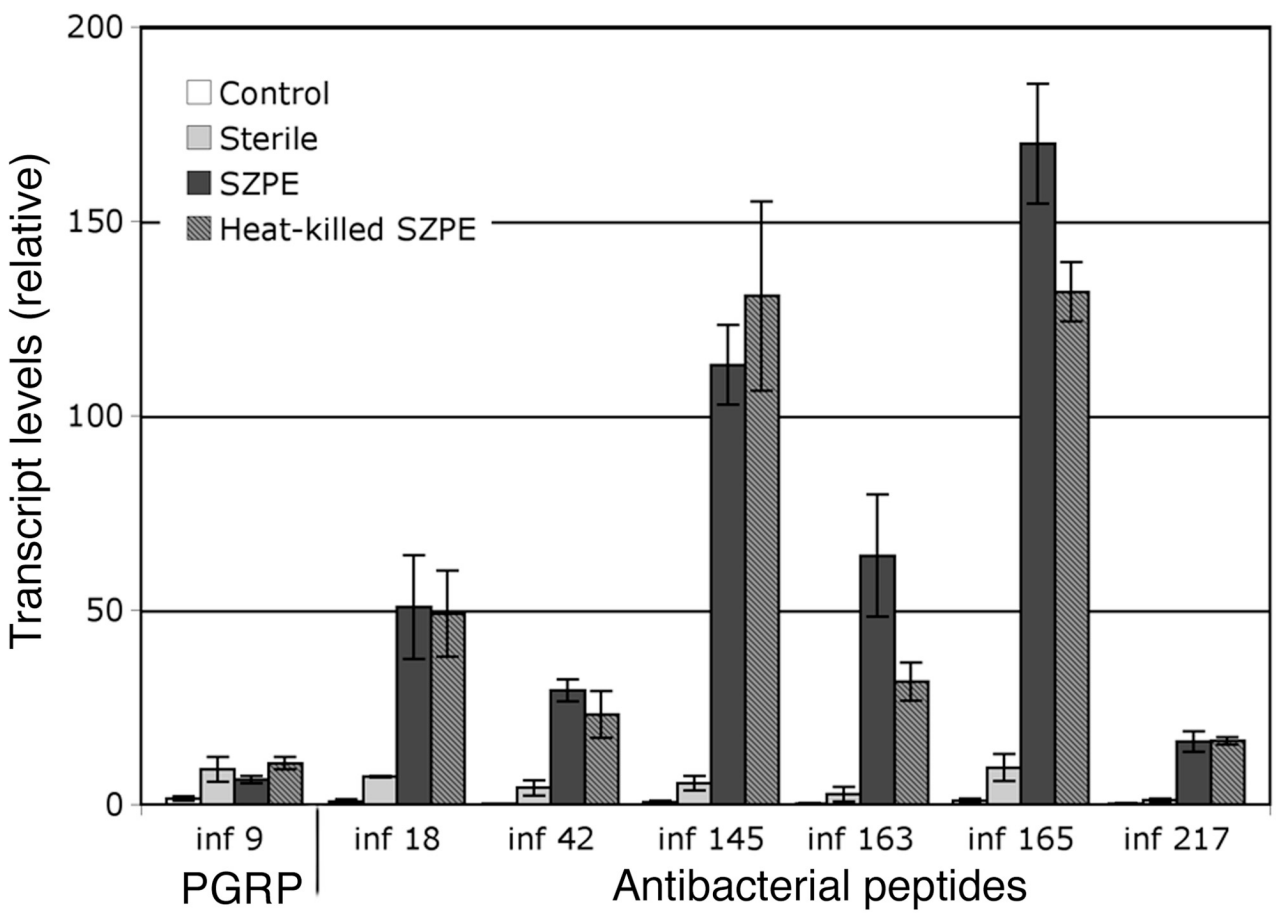
**Analysis of immune gene expression in aposymbiotic larvae challenged with *Sitophilus zeamais *primary endosymbiont**. Transcript of genes induced by an *E. coli *challenge were quantitated by qRT-PCR in untreated aposymbiotic larvae (control) and in larvae six hours after a 69 μl injection of either sterile water (sterile) or SZPE cells (heat-killed or viable) obtained from 50 bacteriomes dissected from symbiotic fourth-instar larvae. ESTs were classified according to sequence similarity as in Table 2. Each bar represents the mean of three independent measurements with standard error.

To examine immune response to SZPE while present in the larval hemolymph, we quantified gene transcript levels in aposymbiotic larvae following an injection of SZPE. Knowing that SZPE fails to divide *in vitro*, we injected approximately 1 × 10^5 ^viable or heat-killed SZPE (Figure 2), the amount of *E. coli *required to induce immune response (data not shown).

No significant difference was shown between water and symbiont injections for *wPGRP1 *(INF-9) whereas all the genes with sequence similarity to AMPs were significantly induced by the symbiont. Moreover, heat-killed *E. coli *was shown to induce a weaker immune response than the untreated *E. coli *(data not shown), possibly because bacterial growth and division in larvae may result in an increase of bacterial density and then in a stronger immune response [16]. Unlike *E. coli*, no difference was found between larvae injected with viable or heat-killed SZPE, which is consistent with an absence of symbiont proliferation in the hemocoel.

### Gene expression analysis in the larval bacteriome

**Figure 3:**
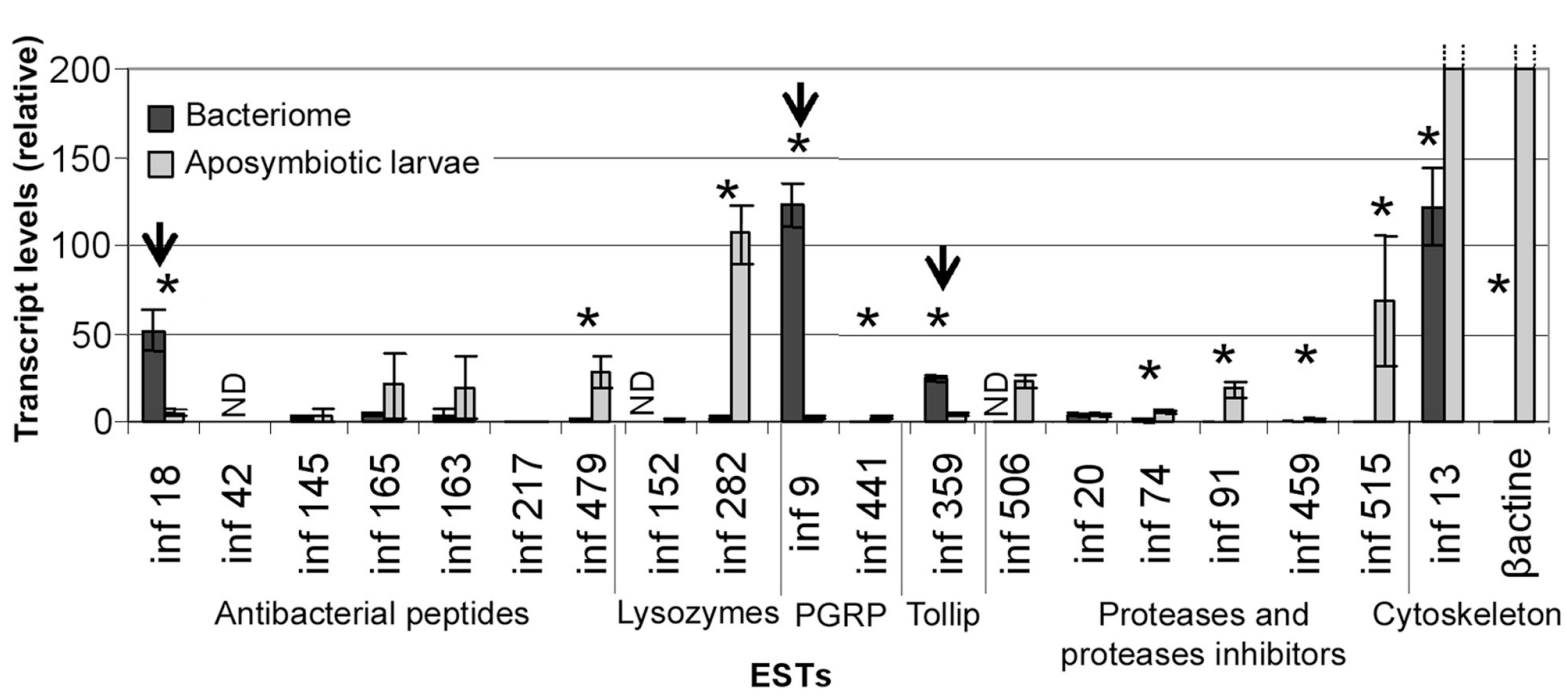
**Analysis of gene expression in the bacteriome tissue**. As described in *Materials and Methods*, transcripts of candidate genes were quantitated by qRT-PCR in whole aposymbiotic fourth-instar larvae (control) and in bacteriomes dissected from symbiotic fourth-instar larvae. ESTs were classified according to sequence similarity as in Table 2. Each bar represents the mean of three independent measurements with standard error. The asterisk represents a significant (*p *< 0.05) difference between the bacteriome and the control, and a significantly high expression in a bacteriome is indicated by an arrow. ND, non-determined.

As the bacteriome fails to develop in the absence of endosymbionts, the immune gene expression profile was examined in the bacteriome and compared with the mean transcript levels from whole aposymbiotic larval tissues. As shown in Figure 3, most of the genes overexpressed in larvae infected with *E. coli *(Table 2) or SZPE (Figure 2) were slightly (or not at all) expressed in the bacteriome, with the exception of two genes: the *wPGRP1 *gene (INF-9; 39-fold) and one of the two *coleoptericin *genes (INF-18; 10-fold). Interestingly, this experiment also revealed a high expression level of the *Tollip *gene (INF-359; 5-fold) in the bacteriome tissue. Moreover, cytoskeleton-encoding genes were shown to be significantly underexpressed in the bacteriome. Actin transcripts were 800-fold less represented in the bacteriome when compared with whole aposymbiotic larvae. Although many cases of host cytoskeleton manipulation by bacteria have been described [28,29], we cannot exclude the possibility that the difference in the levels of actin transcription between the bacteriome and the larvae is due to the high muscle actin content in the larvae. Similarly, the abundance (or absence) of certain gene products in major tissues (for example lysozyme abundance in gut and salivary gland) can create an apparent under (or over-) expression in the bacteriome, and for such genes these results should be considered with caution. However, it is noteworthy that comparisons between the bacteriocyte and the whole body expression profiles have provided previously meaningful results [15,30].

## Discussion

There is compelling evidence that intracellular bacterial infections can establish chronic non-septic relationships with host cells for many generations. Some bacteria (for example, *Chlamydophila *and *Salmonella*) invade and proliferate rapidly in their host, causing diseases, while others (for example, *Mycobacterium tuberculosis*) remain latent and may reactivate months or years after initial exposure to cause chronic disease [31]. In the insect world, some intracellular bacteria can generate a long-term relationship within the host without causing any disease or invading any host tissues except the germ cells and the bacteriocytes. The overexpression of the *wPGRP1 *gene in the weevil bacteriome suggests a close interaction between the host immune system and the endosymbionts.

To determine how the insect immune system recognizes the bacteria and prevents bacterial invasion of insect tissues, we first identified, by SSH technology, the immune genes induced in insects challenged with *E. coli*. Bioinformatic analysis scored a relatively high proportion of ESTs with similarities to immune-related genes, including several antibacterial peptides, lysozymes, *PGRPs *and proteins of the PPO pathway (Table 2), which highlights the efficiency of the subtractive approach. Further *in silico *analysis on full-length coding sequences has confirmed the identification of an i-type and a c-type lysozyme gene (INF-152, INF-282) and at least four antibacterial peptide genes: two members of the coleoptericin family (INF-18, INF-145), a defensin (INF-217) and a homolog of luxuriosin (INF-479), an antimicrobial peptide from *Acalolepta luxuriosa *[20]. We also validated a homolog of *Tollip*, a gene so far only studied in mammals that seems to be absent from the *Drosophila *genome, and a *PGRP *gene (*wPGRP2*, INF-441) additional to the one previously described (*wPGRP1 *gene, INF-9). The expression profile of all the putative immune genes was then characterized by larvae challenge experiments combined with RT-PCR transcript quantification (Table 2). Thus we have demonstrated that all the genes studied were inducible, except for homologs of *Tollip *(INF-359) and *hemolymph proteinase 17 *(INF-459), which appear to be constitutive.

To elucidate the particular bacteriome immune features, we first examined the systemic host response following aposymbiotic larvae injection with SZPE (Figure 2) and we then measured immune gene expression in the bacteriome of naturally infected larvae (Figure 3). Experimental infection of the larvae led to a systemic immune response with the up-regulation of all the genes with similarities to AMPs, providing evidence that SZPE is recognized as a microbial intruder while present in the hemolymph. It is noteworthy that in contrast to SZPE, *Spiroplasma *does not induce host transcripts encoding AMPs in *Drosophila *while present in hemolymph. However, the lack of bacterial cell wall structure of *Spiroplasma *is probably the best explanation for the apparent absence of host humoral response to this bacterium [32].

In the weevil symbiotic larvae, in addition to the *wPGRP1 *gene, one antibacterial peptide induced by bacterial challenge (that is, the *coleoptericin *gene) is overexpressed in the bacteriome. These data confirm the existence of an immune response in the bacteriome tissue and provide the first elements to explain how host-endosymbiont association can persist with regard to the host immune system and bacterial proliferation.

Since all AMP genes are up-regulated following a hemolymph challenge with SZPE, the unique overexpression of the *coleoptericin *gene in the bacteriome suggests a constitutive expression in this tissue rather than an induction by SZPE, unless *coleoptericin *gene regulation involves a separate pathway from those that trigger synthesis of other AMPs. We can only speculate about this question, as comparison between symbiont-full and symbiont-free bacteriocytes cannot be conducted since no symbiont-free bacteriome has been detected in *Sitophilus *aposymbiotic insects so far. Taking into consideration that some AMPs are expressed constitutively in cells potentially in contact with the environment [11,33,34], and that the *coleoptericin *gene encodes a signal peptide, these data suggest that the *coleoptericin *gene is expressed constitutively and secreted outside the bacteriocyte. Coleoptericin is currently under investigation to determine whether this peptide may prevent host tissue invasion by the endosymbiont, and/or may protect the endosymbiont population against infection with environmental bacteria, as the bacteriome tissue is intimately attached to the insect foregut. Moreover, another interesting issue would be to investigate whether *coleptericin *expression in the bacteriome can protect the host from pathogens. This would represent an additional example of 'symbiont-mediated protection' [35].

Although the absence of a host humoral immune response to an intracellular symbiont and a constitutive expression of *coleoptericin *in the bacteriome are consistent with current knowledge of the insect immune response, the overexpression of gene homologs to *PGRP-LB *(that is, *wPGRP1*) and *Tollip *is quite intriguing. *PGRP-LB *gene inhibition in *Drosophila *was indeed shown to result in a significant induction of antibacterial peptides [13], while Tollip has been implicated as a negative regulator of the mammalian immune response [24,25]. Moreover, these two genes are overexpressed in the gut epithelia and have been proposed to play a role in the control of immune reactivity of the host to the presence of bacteria in the gut. Therefore, the overexpression of these two genes in the bacteriome may be consistent with an immune modulation that inhibits the production of AMPs in this organ, as it was described in the gut epithelia. Together with the overexpression of an AMP (that is, *coleoptericin*), these results uncover striking similarities between the bacteriome immune profile and the local immune response in gut epithelium that is in permanent contact with commensal and mutualistic bacteria [9,11,13,33,36,37]. A gene silencing study in weevil is currently under development to validate *coleoptericin*, *wPGRP1 *and *Tollip *gene function and their contribution to the maintenance of symbiosis.

It is noteworthy that this work was performed on a recently established symbiosis where, contrary to ancient symbiotic associations known for a drastically reduced endosymbiont genome size such as in the aphid/*Buchnera *association [38], SZPE exhibits similar features to those of free-living Gram-negative bacteria [39-43]. Hence, whether and how the host immune response evolves in parallel to symbiont genome reduction constitutes a key aspect in the understanding of host-symbiont interaction in the course of evolution. Recently, a transcriptomic study was performed on the aphid bacteriocyte to identify a gene of interest in intracellular symbiosis [30]. Intriguingly, no homolog to any known immune genes was shown to be expressed in the aphid bacteriocyte except for i-type lysozymes, whose function in bacteriocytes remains unknown. Since both the weevil i-type and c-type lysozymes were shown to be weakly expressed in the bacteriocytes, these data suggest that the bacteriocyte immune profile may have evolved with bacterial coevolution with the host, unless the immune response of weevils (holometabolous insects) has diverged significantly from that of aphids (heterometabolous insects).

Whatever the antimicrobial effectors (that is, antibacterial peptides, lysozyme), their permanent expression in bacteriocytes could represent a common mechanism to restrict the localization of mutualistic symbionts, which is probably necessary to optimize the host-symbiont interaction. Without this confinement, intracellular symbiotic bacteria may invade the whole organism without inducing any antibacterial peptide synthesis, as has been shown in *Drosophila *infected by *Wolbachia*, a parasitic symbiont widely distributed in host tissues [44].

## Conclusion

This work provides the first immune gene expression profile in the insect bacteriome and reveals the overexpression of at least three homologs to immune genes: a member of the *PGRP *gene family involved in bacteria recognition, an antibacterial peptide involved in bacterial clearance and a gene involved in immunomodulation in mammals. This immune profile uncovers some striking similarities between the bacteriome and the gut epithelium, which is in constant contact with environmental bacteria. Taking into account that the endosymbiont is recognized as an intruder in the host hemolymph, these findings also indicate that the host immune system may prevent endosymbiont invasion into insect tissues in a manner similar to the gut local immune response that helps to confine the microbiota to the gut, avoiding a permanent systemic response to the commensal bacteria.

## Methods

### Insect rearing

Insects from a SZPE-monosymbiotic strain (*S. zeamais *Lagoa) and from the corresponding aposymbiotic strain were reared and collected as described in Anselme et al. [16].

### Bacterial challenge

Fourth-instar aposymbiotic larvae were challenged by pricking with sterile sharpened needles (mock infection) or with needles previously dipped into a pellet from *E. coli *(TOP10, Invitrogen) overnight cultures, and kept in a moist atmosphere at 27.5°C for 3, 6, and 12 hours. Living larvae were stored at -80°C for RNA preparation. Unchallenged larvae (naive larvae) were treated in parallel as controls.

For the study of the host immune response to SZPE, larvae were injected with 69 nl of either sterile water or bacterial solution containing approximately 1 × 10^5 ^viable or heat-killed (5 min at 95°C) bacterial cells. SZPE solution was freshly prepared from bacteriomes of fourth-instar larvae. Fifty bacteriomes were dissected in buffer A (25 mM KCl, 10 mM MgCl_2_, 250 mM sucrose, 35 mM Tris-HCl, pH 7.5), transferred in a Dounce Teflon Homogenizer and gently crushed in buffer A. After removal of cellular debris by low-speed centrifugation (400 g, 10 min), bacteria were pelleted (10000 g, 5 min) and resuspended to a concentration of approximately 1.45 × 10^6 ^bacteria/μl in buffer A.

### Total RNA isolation

Total RNA from infected and naive larvae was extracted using TRIzol^® ^Reagent (Invitrogen), treated with RNase-Free DNase (Promega) and purified through RNeasy mini kit columns (Qiagen) as described in the manufacturer's procedures. After purification, the RNA concentration of each sample was measured by the Nanodrop^® ^spectrophotometer and total RNA quality was checked by electrophoresis.

### Suppression subtractive hybridization

We applied SSH technology by using a PCR-selected cDNA subtraction kit (Clontech laboratories). For the synthesis of cDNA from *E. coli*-infected larvae used as a tester, three RNA samples were extracted 3, 6 and 12 hours after infection and mixed prior to cDNA synthesis.

Briefly, cDNA synthesis was carried out on a 1 μg aliquot from a mix of 1 μg of each infected RNA sample for the tester and from naive larvae RNA for the driver (SMART PCR cDNA synthesis kit, Clontech laboratories). After phenol-chloroform purification, the infected larval cDNA was digested with RsaI, ligated to two adaptors separately (1 and 2R), then hybridized to RsaI-digested naive larvae cDNA. After hybridization, the subtracted cDNAs were amplified by PCR according to the manufacturer's instructions (Clontech, cDNA subtraction kit). The amplified products were directly cloned into a pCR 2.1-TOPO plasmid (Invitrogen).

### Clone sequencing, Blast homology searching and GO assignment

After transformation by electroporation, around 500 colonies were recovered from Luria Broth agar plates. They were subjected to plasmid extraction (NucleoSpin^® ^Plasmid Kit, Macherey-Nagel) and sequenced in the Institut Cavanilles de Biodiversitat i Biologia Evolutiva (València, Spain). The 485 available sequences were trimmed using SeqClean  to remove flanking vector sequences, adaptors (match with at least 98% identity over at least 11 base pairs) and poly(A/T) tails, and remaining sequences shorter than 60 base pairs were discarded. Because subtracted library construction includes a step of RsaI digestion and adaptor ligation where chimeric cDNA could be formed by cDNA ligation, we checked for RsaI sites in the ESTs and discarded the chimeras from our dataset.

Remaining sequences were clustered using the TGICL assembly program [45] and consensus sequences were conserved for the subtracted library. All sequences (consensus and singletons) were then compared against UniProt using BlastX. For GO assignment, we retained the first hit with at least one GO annotation among the top five hits. GO annotation results were then classified using WEGO [46].

### Sequencing of immune-relevant full-length cDNA

The complete sequences of the transcripts of interest were obtained by 3' and 5' RACE, performed with the SMART RACE cDNA Amplification Kit including the Advantage II PCR kit (Clontech Laboratories). For each gene, the nucleotide sequences of the 3' and/or 5' primers (GSP1 and GSP2) were designed on the corresponding EST (see Additional file 2). The first-strand cDNA used for 5' and 3'RACE were produced by using 1 μg of the infected RNA mix prepared for the SSH method, and using the primers provided in the kit. Amplification of the RACE products was carried out according to the manufacturer's instructions. PCR fragments were gel-purified with the Nucleotrap Gel Extraction Kit (Clontech Laboratories) and inserted into the plasmid vector pCR2.1-TOPO (Invitrogen). The sequences were generated by Genome Express Company (Grenoble, France) with the M13 and the M13rev vector primers.

The full-length sequence of the transcript was predicted using the MacMolly software package according to the RACE PCR product and according to the presence of both in-frame stop codons upstream of the prospective methionine start site and of a poly(A) tail following the prospective stop codon. The predicted sequence was then confirmed by PCR amplification and sequencing. The putative proteins were compared with protein sequences in Swiss-Prot-Trembl to confirm the first homology, and analyzed using the InterProScan  that combines different protein signature recognition methods.

### Real-Time RT-PCR transcript quantification

Real-time RT-PCR transcript quantification was performed with a LightCycler^® ^instrument using the LightCycler Fast Start DNA Master SYBR Green I kit (Roche Diagnostics), as described in Anselme et al. [16]. RNA extractions on three independent biological samples were carried out for each condition (naive larvae, mock-infected larvae and infected larvae) 6 hours after treatment and on samples of 100 bacteriomes dissected from naive symbiotic larvae. After sample purification, reverse transcription into the first strand cDNA was made with the First-Strand Synthesis System for RT-PCR kit (Invitrogen) using oligo(dT) primers. PCR primers were designed on ESTs or cDNA sequences, when necessary, to amplify fragments of less than 300 bp (see Additional file 2).

The PCR reactions were carried out in LightCycler capillaries in a final volume of 20 μl containing 2 μl of cDNA samples (diluted 5-fold), 3.5 mM MgCl_2_, 0.5 μM of each primer and 2 μl of LC-Fast Start Reaction Master SYBR Green I. After 8 min at 95°C, the cycling conditions were as follows: 45 cycles at 95°C for 10 s, primer annealing temperature for 20 s and then 72°C for 30 s. For product identification, a melting curve was constructed at the end of each PCR by heating for 5 s at 95°C and for 15 s at a temperature corresponding to 10°C more than the primer annealing temperature, and then increasing the temperature up to 95°C with increment rates of 0.1°C/s. Reactions were stopped by cooling at 4°C.

For the individual samples, the crossing point (Cp) and, according to the standard curve, the concentrations of the transcripts were determined. The melting curves of each sample were analyzed and the concentrations of samples presenting primer-dimer formation were considered as 'non-determined' (ND). Data were normalized using the ratio of the target cDNA concentration to that of the house keeping gene, *glyceraldehyde 3-phosphate dehydrogenase *(*gapdh*). The expression of this gene is not significantly influenced by the treatments and it is similar to the expression of the ribosomal protein L29 gene (data not shown). Normalized data were analyzed using nonparametric tests.

The sequences of the EST and the full-length cDNA reported in this paper have been deposited in the GenBank database (Accession nos. from EY122775 to EY123248 and from EU282111 to EU282122, respectively). A more detailed analysis on the ESTs (for example, Accession nos., ESTs assembly, Blast results) is available at  (login: sitophilus, password: zeamais).

## Authors' contributions

CA designed and performed experiments, analyzed data (statistics and bioinformatics), wrote the paper and participated in bioinformatic analysis of the subtracted library; VPB participated in molecular studies and sequenced the subtracted library. AV carried out dissections and real-time RT-PCR, and CVM participated in molecular studies and sequence analysis. DC carried out bioinformatics analysis and the URL construction for the subtracted library; AL and AM sequenced the subtracted library and provided critical comments on the manuscript. AH conceived the study, coordinated the work and helped to draft and write the manuscript. All authors read and approved the final manuscript.

## Authors' note

During the preparation of this manuscript, the experimental identification of genes that are induced in response to septic injury in the red flour beetle *Tribolium castaneum *using the SSH method has been published [47]. By comparing EST sequence to *Tribolium *sequence databases, the authors have identified 75 immune-inducible genes in *T. castaneum *potentially involved in immune defense, signaling, and other immunity-linked cellular processes including homologs of, for example, Toll, PGRP-SC, lysozyme, and multiple isoforms of defensins and thaumatin-like peptides. They have also performed a qRT-PCR to determine transcriptional regulation of selected genes (that is, defensin, thaumatin and stress-related genes) in response to either septic versus sterile injury or heat shock.

While this study explored the *Tribolium *immune response considering all cellular processes, we focused our study on *Sitophilus *immune pathways and effectors. It should be noted that, in contrast to this study, we have identified members of different antibacterial peptide families (for example, coleoptericin, defensin). We also identified some homologs of thaumatin-like peptides (for example, INF-475, INF-332, INF-CL57Contig1), but were unable to quantify them without specific amplification.

## Supplementary Material

Additional file 1 **Characteristics of the ESTs from the subtracted library with homology to immune genes.** Click here for file

Additional file 2 **Primers used for 3'- and 5'-RACE (GSP1 and GSP2, respectively) and qRT-PCR.** Click here for file
