# Supplementary material for: Identification of the Weevil immune genes and their expression in the bacteriome tissue
Source: BMC Biol. 2008 Oct 16;6:43. doi: 10.1186/1741-7007-6-43 (PMC2590597; doi:10.1186/1741-7007-6-43)
Supplement: Additional file 1 — Characteristics of the ESTs from the subtracted library with homology to immune genes. [file 1741-7007-6-43-S1.pdf]

Additional File 1: Characteristics of the ESTs from the subtracted library with homology to immune genes.

GenBank Acc. Num[a].	EST[b]	Unigene[c]	CN	Protein description	E-value (Hit)	UniProt Acc. Num.
				Antibacterial peptides		
EY122872	INF-18	INF-CL60Contig1	2	Coleoptericin	3E-15	P80032
EY123087	INF-42	INF-CL6Contig2	6 (+6)[d]	Diptericin A	2.6	Q8WTD5
EY122826	INF-145	INF-CL9Contig1	6	Acaloleptin A	2E-15	Q76K70
EY122846	INF-163	INF-CL13Contig1	4	Cecropin A1	0.68	P81685
EY122848	INF-165	INF-CL5Contig1	15	Sarcotoxin II-1	0.67	P24491
EY122898	INF-217	INF-CL16Contig1	4 (+6)[d]	Tenecin-1	3E-13	Q27023
EY123143	INF-479	INF-CL15Contig1	2 (+2)[d]	Luxuriosin	0.18	Q60FC9
				Lysozymes		
EY122836	INF-152	INF-152-frag1	1	Lysozyme i-1	1E-05	Q6GU90
EY122958	INF-282	INF-282-frag1	1	Lysozyme C-1	6E-17	P00705
				PGRP		
EY123248	INF-9	INF-9-frag1	1	PGRP sb2	7E-57	Q1HRH3
EY123099	INF-441	INF-441-frag1	1	PGRP	9E-38	A0T2Q1
				Immune regulator		
EY123020	INF-359	INF-359-frag2	1	TOLLIP	3E-48	Q9QZ06
				Proteases		
EY122892	INF-20	INF-20-frag1	1	IMPI	2E-11	P82176
EY123240	INF-91	INF-CL19Contig1	3	Cysteine-rich venom-like protein	7E-09	Q5MIW2
EY122936	INF-258	INF-258-frag1	1	Pattern recognition serine proteinase	7E-28	Q69BL0
EY123118	INF-459	INF-459-frag2	1	Hemolymph proteinase 17	3E-10	Q5MPB8
EY123189	INF-515	INF-515-frag1	1 (+7)[d]	Trypsin-like serine proteinase	5E-27	Q64ID5
				Phenoloxidase pathway		
EY123180	INF-506	INF-506-frag1	1	PPAF	2E-15	Q9GRW0
EY123227	INF-74	INF-CL23Contig1	3	Serpin-4A	2E-20	Q6Q2D8
				Cytoskeleton		
EY122818	INF-13	INF-13-frag1	1	profilin	3E-29	Q6QEJ7
-	-	-	-	actin	-	-

[a.] The accession number and [b.] the name of the EST sequence used for RACE and qRT-PCR primer design. [c.] The name of the corresponding Unigene in the database http://mandragore.univ-lyon1.fr/sitozea (login: sitophilus, password: zeamais). [d.] The number of clones corresponding to additional isoforms of the EST is given in brackets. Acc. Num., accession number; CN, copy number: number of clones found in the subtracted library.
