# Supplementary material for: Identification of the Weevil immune genes and their expression in the bacteriome tissue
Source: BMC Biol. 2008 Oct 16;6:43. doi: 10.1186/1741-7007-6-43 (PMC2590597; doi:10.1186/1741-7007-6-43)
Supplement: Additional file 2 — Primers used for 3'- and 5'-RACE (GSP1 and GSP2, respectively) and qRT-PCR. [file 1741-7007-6-43-S2.pdf]

Additional File 2 : Primers used for 3'- and 5'-RACE (GSP1 and GSP2, respectively) and qRT-PCR.

[a.] primer used in a 5'-RACE nested PCR
[b.] primer annealing temperature

RACE Primers	Sequence (5'- 3')
Inf-9-GSP1	TGTTTCTCGGACTTGCCTATGACC
Inf-9-GSP2	CTCTACCAGTCCCTTACGTCGTC
Inf-18-GSP1	GCCATTGATGTTCTTGCCCCAGC
Inf-18-GSP2	TGGGAGGTAAGACCAGACCTGTC
Inf-42-GSP1	CCAACACGTTTGTCACCGGTGTG
Inf-152-GSP2	GGACGCCAGTCAGGTGACTCTAC
Inf-165-GSP1	CGTGGTCAGCACCAACGCCCAAA
Inf-165-GSP2	CATCACCTCGACGGTTCTGGCTA
Inf-217-GSP1	AATGGGCCGCACAAGCTGAATCG
Inf-282-GSP1	GAGAATTCCGTAGTCGCCGGTCT
Inf-359-GSP2	CACCTACGGACCCTAATGGTGGC
Inf-441-GSP1	CCCAACCCTTCTGGTCCATGTGG
Inf-441-GSP2	CCTTCTGCTGCACAGTTGACGGC
Inf-441-GSPN2[a]	CAGCAACTGCCTGCCCTGGTGAC
Inf-479-GSP2	GGGTCTCACGGCGAATACAGAGAC

qRT-PCR Primers	Sequence (5'- 3')	T°C [b]	qRT-PCR Primers	Sequence (5'- 3')	T°C [b]
5'inf-9	ATAATTTCGCTGTTGGAGGG	58	5'inf-165	AGTCACAAAGGAACAATTTTGG	58
3'inf-9	TCTCGGACTTGCCTATGACC		3'inf-165	AGAAGCCGTCACGTCCGTTC	
5'inf-13	GAAGAGGTGAGGATGTCCT	58	5'inf-217	ACGCTGTAGTGATAAGTGCA	58
3'inf-13	ACATGAGTTGGCAGGATTAC		3'inf-217	CACACGTACAGGTCCTTCT	
5'inf-18	GAATAGATACAACGGGGGTCA	58	5'inf-282	GCGGGGAAAAAAAGTGGCGTA	58
3'inf-18	CTACCATCTGACACTTCCTC		3'inf-282	CCATCGGGATTGTGGTTTTTAG	
5'inf-20	ACCCTGGACCTAACGATCC	58	5'inf-359	AAGAGCGACGCAATAGGGT	58
3'inf-20	TACTGCTACAAACGAACATTG		3'inf-359	CATTAGGGTCCGTAGGTGT	
5'inf-42	TCAGGATGAAGATGGCCAAG	58	5'inf-441	TGCAAGGCCTGCCCTTAGT	58
3'inf-42	ACACTGGTACTCTGGCATCT		3'inf-441	GTGCCATCTCCTCCAATCAT	
5'inf-74	TCTGTTGTATGGCATTCCGA	58	5'inf-459	CAACAAATCCAGACGAGCC	58
3'inf-74	AGCGCTTGAAGTATCGAACT		3'inf-459	ACCACCGCATAGCCATCG	
5'inf-91	GATCGGTATACTTCAGCGAA	58	5'inf-479	ACCTTCACGACCCACACAT	58
3'inf-91	AACGCATCTACCAGTTCTGT		3'inf-479	GTCATACGATACGTTTTCAGT	
5'inf-145	TCGGCATCCCCTAATCCAGAC	64	5'inf-506	CAATCGTTTATGGTCAGGAC	58
3'inf-145	CTACCAGCGGATGGCGCCACC		3'inf-506	GGAATTCCACAACAAACGTC	
5'inf-152	CTCTCCGTCACCTCGTGTTAT	58	5'inf-515	GGTTCGAACGCCGTCAGA	58
3'inf-152	TCCTGCTCGTTCTTTGTCAT		3'inf-515	TCACGGAGATGTCGTTAGC	
5'inf-163	GTCCTGTTAGTTGTGGCAGTA	58	Actin-for	CAACTTCCCTAGAAAAGAGC	60
3'inf-163	TTATCTTCCAGGCACCAATCC		Actin-rev	TTCCTTCTGCATCCTGTCGG	
			Gapdh-for	AACTTTGCCGACAGCCTTGG	58
			Gapdh-rev	GCGCCCATGTATGTAGTTGG	
